# Management Outcome and Associated Factors of Necrotizing Soft Tissue Infections in an Ethiopian Tertiary Care Hospital: A-Five-Year Review

**DOI:** 10.4314/ejhs.v34i5.4

**Published:** 2024-09

**Authors:** Esubalew T Mindaye, Fitsum Terefe

**Affiliations:** 1 Department of Surgery, SPHMMC, Addis Ababa

**Keywords:** Soft tissue infection, necrotizing fasciitis, gangrene, significant surgical infection

## Abstract

**Background:**

Necrotizing soft tissue infection (NSTI) is one of the deadliest diseases among surgical infections. Prompt timely diagnosis and urgent surgical intervention with supportive care are cornerstones of patient management. This study aimed to assess patient outcomes and associated factors of adult patients diagnosed and surgically treated for NSTIs at Saint Paul's Hospital Millennium Medical College (SPHMMC), Ethiopia from January 2015 to December 2019

**Method:**

An institution-based cross-sectional study was conducted by reviewing medical records of patients treated for NSTIs at SPHMMC in the 5 years study period. A five-section survey instrument was developed, and the collected responses were cleaned and entered into Epi data (v3.1) and exported to SPSS (v.26). Statistical analysis of associated factors was done with binary logit regression model.

**Result:**

Medical records of 110(84%) patients were retrieved and nine out of ten subjects were male with a median age of 42 years (IQR- 34-62yrs) The leading clinical presentations were painful swelling 96(87.3%), fever 79(71.8%) and foul-smelling discharge 62(56.4%). Five out of ten participants have known comorbidity and 9 out of 10 patients have specified predisposing events before their infection. The majority (86.3%) underwent surgical debridement and amputation was done for eighteen patients. The average length of hospital stay was 27 days (2 to 112 days range) with mortality rate of 20%. advanced age, shock at presentation, post-operative anemia, and infection involving the torso were significantly associated with poor patient outcomes.

**Conclusion:**

Surgical management of NSTIs has favorable result and patient presentation and anatomical location of the lesion determine patient outcome.

## Introduction

Necrotizing soft tissue infections (NSTIs) are infrequent, but deadly infections associated with higher treatment cost and scarce resource utilization. They commonly occur around the abdomen (18-64%), perineum (36%) and lower extremity (36-55%) but no anatomical area is spared from being involved (1–5). They are characterized by significant necrotic changes and involve any of the layers of dermis, subcutaneous tissue, superficial or deep fascia or even the underlying muscle (6,7). Although it has been described since the time of Hippocrates around 500 BC, clear clinical definition and classification of NSTIs is still ambiguous leading to confusion among clinical practitioners in addition to rarity of the disease (4,5,8).

Insect bites, illicit drug injection sites, perianal abscesses, trauma sites and surgical procedures in immunocompromised or local tissue hypoxia states, corticosteroid use, diabetes mellitus, chronic alcoholism, malnutrition, burns, cancer or peripheral arterial disease are common predisposing events before NSTI; but up to 30% of patients have no identifiable risk factor (2,3).

The infection spreads rapidly through the soft tissue planes and produces severe systemic sepsis leading to septic shock, multiple organ failure, and death unless early aggressive treatment is instituted (1,4).

Based on the microbiological characteristics, NSTIs are broadly classified into Type I, which is poly-microbial and accounts for 55–75% of infections; Type II, mainly caused by Group A beta-hemolytic Streptococci and accounts for 10-15% of NSTIs; Type III, caused by Cl. Perfringens resulting clostridial gas forming myonecrosis and accounts for < 5% of all NSTI; Type IV, extremely rare infection caused by Vibrio vulnificus and fungi (2,3,7,9). Necrotizing fasciitis, one of the diseases mentioned under NSTI, is a devastating acute infection with a high morbidity and mortality rate. It is a potential limb and life-threatening disease, which mainly involves the soft tissues (2).

Rapidly progressing pain, fever, anxiety, and diaphoresis which is out of proportion to local signs of trauma are classic symptoms associated with NSTIs. Patients might have a history of trauma or a break in the skin within 48 hours before the onset of symptoms, but it is quite rare accounting for only 10% to 40% of patients' classic presentations (7). Diagnosis is mainly clinical; however, it is often difficult to diagnose NSTI early, and sometimes patients are treated as simple cellulitis until they rapidly deteriorate (8,9). Antibiotic therapy is mandatory, and early surgical exploration and debridement are critical to ensure a good outcome(9).

The success of treatment for NSTIs rests on early recognition, adequate resuscitation, broad-spectrum antibiotic therapy, radical surgical debridement, and supportive care. Surgery aims to debride all necrotic soft tissue and fascia to prevent the progression of the disease and aid speedy recovery. Despite all these mortality is reported to be 30-60%, and age >60, being female or having chronic heart disease, cirrhosis, skin necrosis, pulse rate >130/min, systolic BP <90 mmHg, and serum creatinine ≥1.6 mg/dl were identified as poor prognostic factors (8,10).

The study aimed to determine the in-patient management outcomes of surgically treated NSTI patients at Saint Paul's Hospital Millennium Medical College (SPHMMC). It also further explored variables associated with patient morbidity and mortality. The results of the study will contribute to the development of management protocols and to provide a benchmark for further studies on the area.

## Materials and Methods

The survey was conducted at St. Paul's hospital millennium medical college (SPHMMC) which is the second-largest multi-specialty tertiary care teaching hospital in Ethiopia. The hospital has about 100 in-patient beds and 5 Major OR tables dedicated to General Surgery Department. The hospital carries out about 5000 surgeries annually. Surgical procedures related to NSTIs are performed by consultant General Surgeons or Surgical residents under supervision (11). The cross-sectional study was conducted from April 1, 2020 – May 30, 2020 G.C, by reviewing medical records of adult (>15years) patients who undergone inpatient surgical management for the diagnosis of necrotizing soft tissue infections at SPHMMC over the 5 years' study period of January 1^st^, 2015 to December 31, 2019. Patients with lost medical records and those who were treated and referred from other health facilities for skin graft and reconstructive surgery were excluded. The study aimed to assess inpatient management outcomes and associated factors of NSTIs.

**Sample size determination and sampling technique**: As Necrotizing soft tissue infections (NSTIs) are rare surgical disease all patients surgically treated for NSTIs at SPHMMC during the study period were included. During the study period 131 patients were diagnosed and surgically treated for NSTs out of which the medical records of 110 patients were retrieved.

**Data collection and scoring**: Data was collected by trained general practitioners using a *five-section* semi-structured questionnaire that was prepared after a thorough literature review. Patients' medical records were reviewed for patient demographics, comorbidities, clinical and laboratory features at initial presentation, wound, and blood culture results, treatment, the extent of surgical debridement, and patient outcomes Laboratory Risk Indicator for Necrotizing Fasciitis (LRINEC) score was assessed from laboratory results available in medical records. The questionnaire has five major components: Demographic data, Predisposing factors, Clinical presentation, Laboratory variables, and Management outcomes.

**Dependent variables:** Outcomes of the treatment (died or discharged with improvement).

**Independent variables:** socio-demography(age, sex, address, marital status), predisposing factors(post-traumatic, post-surgical, perianal lesions, urethral stricture or post-urethral instrumentation, boils and Furuncles, chronic wound or ulcer), Clinical presentation (duration, presenting clinical features), laboratory investigation (WBC, Hemoglobin and HCT, Creatinine and BUN, Serum electrolyte, Culture and sensitivity, Serum glucose level, C reactive protein), co-morbidity and treatment given

**Data processing and analysis**: The processing begun by checking the gathered data for accuracy and completeness. Each completed questionnaire was assigned a unique code and entered into a computer by using *Epi data (v3.1)* and exported to SPSS version 26.00 (IBM, Armonk, NY, USA) for further analysis. Associations were analyzed by binary logit regression model. Results are presented as frequency distributions, cross-tabulations, and graphs. Continuous variables are presented as mean and standard deviation (SD) and categorical variables as frequency and percentages.

**Outcome of the treatment**: died or discharged with improvement.

**Ethical clearance:** Clearance was obtained from the Institutional Research and Ethics Review Committee (IRB) of the SPHMMC. The collected information will only be used for this study and will be kept confidential.

## Results

**Socio-demographic characteristics**: A total of 131 patients were admitted and treated for NSTIs at SPHMMC during the five-year study period. Of these, 110 (84%) case records were retrieved and included in the study. The median age of the patients was 42 years (IQR-28) and majority 40 (36.4%) were in the age group of 30-44 years. Eight out of ten participants were male and about 59 (54%) of them were from urban area. Majority of the patients 75 (68.2%) were married.

**Predisposing factors, comorbidities, and clinical presentation of NSTI patients**: Nine out of the participants had specified predisposing factors for NSTIs, the most common being perianal lesions 23(20.9 %) followed by chronic wound/ulcers and urethral stricture 14(12.7% each). Half of the participants had known comorbidities Diabetes Mellitus 20(18.2%), HTN 18(16.4%) and Peripheral arterial disease 12(PAD) (10.9%) being the leading once.

**Figure 1:**
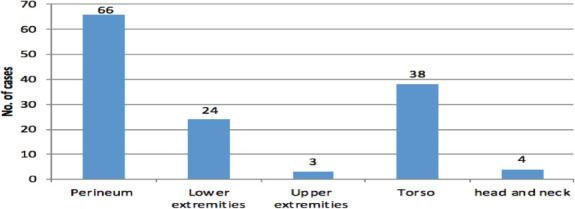
Anatomic site involved in patients treated for NSTIs at SPHMMC Addis Ababa, from January 1st, 2015 to December 30, 2019

Seventy-eight (71%) of the participants presented within a week of the onset of their illness. Pain full swelling 96(87%), fevers 79(72%), and foul-smelling discharge 62(56%) were the most common clinical presentations (Table 2). The most common anatomical location involved was the perineum followed by the torso and lower extremities (Figure 1).

**Table 2:** Modes of management given for patients treated for NSTIs at SPHMMC Addis Ababa, January 2015 to December 2019

Characteristics	Category	Frequency	Percent
Number of Debridement sessions	1×	44	46.3
	2×	31	32.6
	3×	12	12.6
	4×	6	6.3
	5×	2	2.1
Site of amputation	Lower Limb	17	15.5
	Upper Limb	1	0.9
Mode of Wound Closure	Delayed primary closure	43	48.9
	Secondary Closure	14	15.9
	Transfer to plastic surgery	30	34.1
	primary closure	1	1.1
Length of hospital stay	1-7 days	18	16.4
	8-14 days	21	19.1
	15-21 days	18	16.4
	22-28 days	12	10.9
	Above 28 days	41	37.3

**Surgical management and hospital stay**: Surgery was the mainstay of treatment for all patients involved in the study and 95(86.3%) of them have undergone surgical debridement while 18 (16.3%) patients required amputation. More than half of the participants required multiple debridement sessions (≥2 sessions) and stayed for more than 3 weeks in the hospital before being discharged.

**Post-operative Out come and laboratory profile**: Seventy-five percent of the participants have uneventful smooth post-operative course while the rest have developed complication the leading once being wound site infection 27(24.5%), post-operative anemia 20(18.2%) and septic shock19 (17.3%). Eighty percent of the patients were discharged with improvement while the rest 22(20%) succumbed due to their illness.

LRINEC score was not calculated. The average value of other parameters of the index (serum sodium, hemoglobin, white cell count, creatinine and glucose) are deranged as presentation of the patients is around 10 days on average.

### Analysis of factors associated with surgical management outcome

#### Binary logit regression mode 1

**Univariable analysis:** Univariable binary logit regression is fitted for the outcome of patients to see the effect of each explanatory variable. Infection involving the torso, and post-operative anemia have a significant effect on the outcome of patients when fitted separately at a 5% level of significance.

**Multivariable Analysis**: We checked the association between the explanatory variables and the outcome of patients in the univariable binary logit. Then we took the variables which are significant at a 10% level of significance. We used forward variable selection method to get the final binary logit model that best fits to our data

Our study showed that older age, Post-operative anemia, Shock at presentation, and Infection site involving the torso were significantly associated with poor management outcomes of patients with NSTIs.

## Discussion

Although it's very challenging to get the exact figure in resource-limited countries like Ethiopia, the incidence estimated in the USA is 0.04 per 1,000 person-years, and in the United Kingdom 500 cases per year(12). Delay in diagnosis and management is associated with higher mortality ranging from 6-36% in different studies which is comparable with the result in our study 22(20%) (10). There is no evident gender and age predilection worldwide, but a significant number of the participants 91(82%) involved in our study were male and the majority of them were above the age of 30 years. Nearly half of the patients were from urban areas.

About 89% (n=98) of the participants have at least one identified predisposing factor for the development of NSTI, the most common being perineal lesions followed by chronic ulcers due to peripheral arterial disease and urethral stricture. In this regard, Yilma et. al reported the presence of predisposing factors in 39.7% (n=31) of his patients the most common being perianal fistula and abscess (13). However, Hodgins N. et al. identified no recognizable mechanism of infection initiation in 37/46 of his patients(14). Considering the number of patients involved in our study, the number of patients with identifiable predisposing factors were significantly higher than those reported in the above studies.

Half of the patients involved in our study have known comorbidities with Diabetes mellitus, HTN, and peripheral arterial disease being the leading ones and this was comparable with the findings by G. Singh et al (2002) and, Anaya and colleagues(2005), Diabetes mellitus being the most common associated illness (n = 22, 29%) and 19% (n=32) respectively(15).

About 71% of the participants presented to a health facility within a week after the onset of their illness and the overall mean duration of the presentation was 10 days which is comparable with Jacob Ndas Legbo's report of 9.9 days from Nigeria (8) but it was late when compared with 3.4 days of Nissar Shaikh et.al, Doha, Qatar (2). This might be due to poor health-seeking behavior in low-income countries.

Painfull swelling 96(87%), fever 79(72%), foul-smelling discharge 62(56%), and skin necrosis 55(50%) were the leading clinical symptoms of our patients, a finding comparable with the report by N. Hodgins et.al. 2014(14). Hypotension on presentation (systolic pressure <90 and diastolic pressure < 60mmhg) was found, in only 22.7% (n=25) and 35.5% (n=39) of the participants respectively. A systolic pressure of <90 mmHg at presentation was associated with increased mortality comparable with report of Goh.et.al (16).

Early Surgical debridement targeting on expeditious removal of all necrotic and infected tissues has shown to improve outcomes in patients with NSTIs (5). Optimally, an average of three debridement sessions which spaced 12 to 36 hours apart is needed to obtain control of gross infection (4). In these regard, 95(86.3%) of our patients have undergone surgical debridement which ranged from 1 to 5 sessions and majority (53.7%) have required multiple debridement sessions with median of 2 sessions and was similar with the report by yilma.et.al.(13). The magnitude of limb loss in our study was 16.3% (n= 18).

NSTI is a surgical urgency with a high mortality rate, even with sufficient treatment, with the reported rate of mortality varying from 6% to 36% (10). The mortality rate in our study 20% is comparable with report by Khamnuan et al (Thailand) of 19.3% (290 patients of 1,504 patients) (10) and by Yilma et. al.(Jimma, Ethiopia) of 19.23% (n=15) (13).

Patients recovering from NSTIs have higher morbidity and require a prolonged recovery period. The median length of hospital stay in our study was 20 days which ranged from 2 to 112 days. One-fourth of our patients had developed post-operative complications, which included wound infections 27/110, post-operative anemia 20/110, septic shock 19/110, and multi-organ failure 15/110. Postoperative anemia was significantly associated with NSTI mortality.

In conclusion, late patient presentation with hemodynamic instability was found significantly associated with higher morbidity and mortality rates. Most participants had preceding perineal lesions which could have been treated earlier avoiding this fatal consequence. Healthcare stakeholders should take a lesson to deliver health education to improve patients' presentation to health facilities and incorporate NSTI patient care as a surgical quality improvement project. None of our patients had culture and sensitivity tests and C-reactive protein tests, which would have helped guide patient management and, predict mortality and morbidity. As a result of resource limitations, the serum level of C-reactive protein, which is the major component of the LRINEC score, was not determined in all of our patients.

## Figures and Tables

**Table 1:** Predisposing factors, comorbidities and Clinical presentation of patients treated for NSTIs at SPHMMC, Addis Ababa, Ethiopia, from January, 2015 to December, 2019 G.C.

Characteristics	Category	Number	Percent (%)
Presence of Predisposing factors	Present	98	89.1
	Absent	12	10.9
Predisposing factors	perianal lesions	23	20.9
	Trauma	7	6.4
	Chronic wound/ulcer	14	12.7
	boils/furuncles	10	9.1
	Post injections	4	3.6
	postoperative wound infections	10	9.1
	Urethral stricture	14	12.7
	vulvar abscess	5	4.5
	After herbal medicine application	2	1.8
	Others*	9	8.2
Co-morbidities	Present	55	50
	Absent	55	50
Type of Co-morbidities	Diabetes Mellitus	20	18.2
	HTN	18	16.4
	PAD	12	10.9
	Malignancy	4	3.6
	RVI	2	1.8
	Others^a^	12	10.9

Clinical presentation patterns					

	Sign and symptoms			Frequency	Percent
	Duration of illness at presentation	≤1 week		78	70.9
		>1 week		32	29.1
**Clinical presentation**	Pain full swelling Foul smelling discharge			96	87.3
				62	56.4
	Fever			79	71.8
	Skin necrosis			55	50.0
	Darkening of extremities			16	14.5
	Palpable crepitation			12	10.9
	Pulse rate	<100 bpm		36	32.7
		<100 bpm		74	67.3
		Systolic	<90mmhg	25	22.7
	Blood pressure at presentation		≥90 mmhg	85	77.3
		Diastolic	<60mmhg	39	35.5
			≥60 mmhg	71	64.5

* *Post angioplasty, suprapubic needle aspiration, skin lesion, AV fistula site for dialysis, peri colostomy wound infection, infected Hydrocele, dental abscess, post iodine burn, emphysematous pyelonephritis*

a *Cardiac disease, stroke, asthma, CKD, Psychiatric Disorders, preeclampsia, cerebral palsy, and Paraplegia*

**Table 3:** Postoperative complications and management out comes of patients treated for NSTIs at SPHMMC Addis Ababa, from January 1st, 2015 to December 31, 2019, G.C

	Characteristics	Frequency	Percent
Post operative complications	Wound Infection	27	24.5
	Septic shock	19	17.3
	Multi-organ Failure	15	13.6
	HAI	10	9.1
	Respiratory Failure	10	9.1
	Post-operative Anemia	20	18.2
	DVT	5	4.5
	Urethro-cutaneous fistula	6	5.5
	Others*	2	1.8
	Improved	88	80.0
Condition at discharge	Died	22	20.0

* *Others: Myocardial infarction, DKA*

**Table 4:** Univariable Analysis of factors associated with surgical management outcome

Variable	Covariates	Sig. (P-value)	Exp(B)	95% CI
Demographic factor	Age	0.062	1.027	0.999-1.055
	Place of residence	0.091	0.425	0.158-1.146
Clinical presentation	Systolic hypotension at presentation (<90mmhg)	0.022*	3.277	1.186-9.052
	Diastolic hypotension at	0.037*	2.769	1.063-7.211
	presentation (<60mmhg)			
	Tachycardia at presentation	0.119	0.394	0.122-1.270
	Duration of illness at	0.786	1.156	0.406-3.286
	presentation			
Anatomic site involved	Infection site involving the Torso	0.011*	0.284	0.108-.748
Postoperative	Presence Post-op anemia	≤0.001*	0.114	0.039-0.337
Complications	Presence of post-op wound infection	0.410	0.649	0.232-1.814
Laboratory factors	WBC count at presentation	0.894	1.000	1.0
	Hemoglobin at presentation	0.056	0.835	0.693-1.005
	Pulse rate at presentation	0.230	1.017	0.989-1.046

**Table 5:** The final binary Logit model result for factors associated with poor management out come

Variables	Sig.	OR	95% CI
AGE (continuous.)	0.016*	1.045	1.008-1.082
Post op anemia(present) Ref (absent)	0.001*	9.644	2.769-33.582
shock at presentation (systolic blood pressure) (<90mmhg), Ref (≥ 90mmhg)	0.045*	3.446	1.028-11.558
Infection involving Torso (Yes), Ref (No)	0.011*	4.780	1.426- 16.017
Constant	0.000	0.006	
